# Buffer Influence on the Amino Acid Silica Interaction

**DOI:** 10.1002/cphc.202000572

**Published:** 2020-09-23

**Authors:** Saientan Bag, Stefan Rauwolf, Mikhail Suyetin, Sebastian P. Schwaminger, Wolfgang Wenzel, Sonja Berensmeier

**Affiliations:** ^1^ Institute of Nanotechnology (INT) Karlsruhe Institute of Technology (KIT) Karlsruhe Germany; ^2^ Bioseparation Engineering Group Department of Mechanical Engineering Technical University of Munich(TUM) Garching Germany

**Keywords:** amino acids, buffer, chromatography, multiscale modelling of adsorption, silica

## Abstract

Protein‐surface interactions are exploited in various processes in life sciences and biotechnology. Many of such processes are performed in presence of a buffer system, which is generally believed to have an influence on the protein‐surface interaction but is rarely investigated systematically. Combining experimental and theoretical methodologies, we herein demonstrate the strong influence of the buffer type on protein‐surface interactions. Using state of the art chromatographic experiments, we measure the interaction between individual amino acids and silica, as a reference to understand protein‐surface interactions. Among all the 20 proteinogenic amino acids studied, we found that arginine (R) and lysine (K) bind most strongly to silica, a finding validated by free energy calculations. We further measured the binding of R and K at different pH in presence of two different buffers, MOPS (3‐(*N*‐morpholino)propanesulfonic acid) and TRIS (tris(hydroxymethyl)aminomethane), and find dramatically different behavior. In presence of TRIS, the binding affinity of R/K increases with pH, whereas we observe an opposite trend for MOPS. These results can be understood using a multiscale modelling framework combining molecular dynamics simulation and Langmuir adsorption model. The modelling approach helps to optimize buffer conditions in various fields like biosensors, drug delivery or bio separation engineering prior to the experiment.

## Graphical abstract

**How do buffers affect protein surface interactions**? Experimental zonal elution chromatography helps to identify interactions of silica surfaces with amino acids. These interactions can be comprehended with a multiscale modelling framework combining molecular dynamics simulation and different flavors of the classical Langmuir adsorption model.

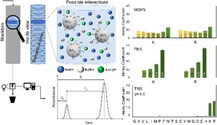

## 1 Introduction

Protein and peptide‐surface interactions at the solid‐liquid interface play an important role in various research fields like medicine,[^1^, ^2^] engineering^[3]^ and biotechnology.^[4]^ These interactions depend on the detailed structure and the composition of both surface and protein. However, it is difficult to characterize these interactions for small peptides or individual amino acids (AAs). There have been a number of studies that investigated such interactions for metal, metal oxide, polymer or silica surfaces both experimentally and theoretically,[^5^, ^6^, ^7^] but trends for peptides have been difficult to derive due to complexities of the composition of the system.^[8]^ The latter comprises not only the peptide and the surface but also the solvent, which often contains a buffer to stabilize the pH of the system. The fact that buffer ions can compete with the peptide/ protein in binding to the surface is well known and investigated,[^9^, ^10^, ^11^, ^12^, ^13^, ^14^, ^15^, ^16^, ^17^, ^18^, ^19^, ^20^] but to the best of our knowledge this has never been done for single amino acids especially for silica. A rational understanding of peptide or protein interactions with surfaces would benefit greatly from data on the interactions of individual amino acids. Calculations of such interactions are often complicated by the lack of adequate models that describe the surface and its interactions with the amino acids. For this reason, we study here silica surface, which is widely used in various applications and for which a pH dependent model has been recently developed.^[21]^ In an earlier work, Rimola et al.^[22]^ tried to quantify the adsorption affinity of 15 AAs on silica by calculating the adsorption energy using ab INITIO ONIOM2 within a cluster approach. A heuristic entropy correction was made to obtain the adsorption free energy from the adsorption energy.

There are many different ways to study the interaction between amino acids and surfaces like silica, such as spectroscopic methods or controlled bind and release experiments, which have been already discussed in depth in several reviews.[^5^, ^6^, ^7^] Most studies focus on glycine and alanine, which are the most simple structured molecules having both, an amino and a carboxy functional[^23^, ^24^, ^25^, ^26^, ^27^, ^28^] group. Only few comparative studies between different amino acids, pH and ion strength exist.[^29^, ^30^, ^31^, ^32^, ^33^, ^34^] Most of the AAs do not interact with the silica surface in aqueous systems because they are present in their zwitterionic form in which the attraction of NH^3+^ and repulsion of COO^−^ groups by the silanol groups balance each other.^[32]^ Only AAs with additional charged groups, e. g. arginine, lysine and histidine can interact electrostatically with the negatively charged silanols on the silica surface.[^32^, ^35^] These interactions are highly pH dependent. A significant interaction can be observed at pH>5 which can be explained through appearance and accumulation of negatively charged groups on the silica surface^[32]^ at pH>5. The electrostatic interaction can lead to the formation of outer‐sphere complexes, where positively charged amino acids coordinate to the silica surface.^[32]^ There are also studies indicating that hydrogen bonding between silanols and the functional groups of the amino acids has a huge influence on amino acid adsorption at very high pH (pH>10).^[28]^ The third contribution to adsorption of amino acids are hydrophobic interactions with the Si−O−Si surface groups of silica as shown for phenylalanine or benzene as aromatic molecules.[^33^, ^36^] But with higher pH the influence of hydrogen bonds and hydrophobic interactions diminish due to the increasing electrostatic interactions.^[37]^

Chromatography, which usually exploits the different interaction strengths of various components in a mixture with a surface is widely used for analytical purposes and for the purification of biopharmaceuticals.^[38]^ Although in high‐performance affinity chromatography, zonal elution is one of the most common formats to study biomolecular interactions, it is yet rarely used for interaction studies of amino acids with silica. Zonal elution is performed by injecting a small volume of analyte onto a column under isocratic conditions and by monitoring the elution time. The elution time of the target is directly related to the target's interaction strength with the resin. These experiments are performed with different conditions, which lead to detailed information about the nature of interactions. Conditions that can be altered are pH, ionic strength, temperature, composition of the mobile phase.[^39^, ^40^, ^41^] Basiuk and coworkers were the first to use chromatographic retention data to obtain free adsorption energies for single AAs on silica in water.[^29^, ^30^, ^31^] Here we extend this work to determine the strength of the interactions under different conditions by measuring the time an analyte needs to pass through the column in relation to a non‐binding analyte.[^42^, ^43^, ^44^] In order to understand the binding behavior of peptides and proteins, the natural AAs serve as a useful reference to improve the understanding of the protein and peptide‐surface interactions with the stationary phase.[^6^, ^45^] As the use of buffer is essential in biotechnology the AA‐surface interaction should also be investigated in presence of the buffer.^[38]^ However, a detailed understanding of the buffer influence is required to make results transferable between systems. In this paper, we demonstrate the strong influence of buffers on AA interaction with silica combining experimental and theoretical methodologies. We perform the column chromatography zonal elution experiment to determine the Henry coefficient, which is a descriptor for the propensity of binding events between solid and liquid phase in chromatography.^[46]^ The interaction of all the 20 AAs with the silica solid phase were investigated in presence of different buffers. We also formulated a multiscale modelling framework combining molecular dynamics (MD) simulation and different flavors of Langmuir model to understand the amino‐acid adsorption in presence of different buffers. MD simulation was performed to evaluate the energetic parameters of the adsorption of a single molecule which is further used in mechanistically different Langmuir models to predict adsorption behavior of thermodynamically large numbers of adsorbate molecules in different physical conditions.

## 2 Results and Discussion

### 2.1 Amino Acid Binding in Aqueous Solution

**Figure 1:**
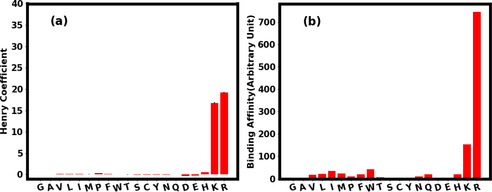
a) Henry coefficient for the binding of AAs to silica in 10 mM TRIS pH 8 as measured [see Equation (1)] using the chromatography experiment. b) Binding affinity of the AAs in water calculated using umbrella sampling simulation [see Equation (2)]. The positive charged AAs, R and K bind strongly to silica as found in both experiment and simulation.

We first measured the interactions between AAs and silica solid phase in a chromatographic system in aqueous buffered solution (Figure 1a). The retention factor *k_i_* of the amino acids is measured (see Experimental Section) in relation to a non‐binding analyte (in this case Uracil) and converted into the Henry adsorption coefficient *H* (see Table S6 in Supporting Information). In chromatography, retention factor of 1 means a slight interaction with the column. A retention factor of 20 means strong interactions because the analyte is spending a lot of time interacting with the resin. Retention factors >20 are problematic because this means extreme long run times and poor sensitivity due to peak broadening.^[47]^ The Henry coefficient is directly related to the retention factor only multiplied by the phase ratio of the column [see Equation (1)] for better comparison of different packing. As the phase ratio of the columns is between 0.7 and 0.9 the values can also be applied for the Henry coefficient. The Henry coefficient (*H*) is the linear equilibrium constant between solid and liquid phase in chromatography and can thus be used as the equilibrium constant *K* in the van't Hoff equation which gives information about the difference in free energy of adsorption.^[30]^

(1) $$\ln k_{i}=\ln K+ln\frac{V_{S}}{V_{M}}=\frac{-\Delta G^{0}}{RT}+ln\frac{V_{S}}{V_{M}}$$

Here $\Delta G^{0}$ is the free energy of adsorption. *V_s_* and *V_m_* are the phase ratio between the volumes of the stationary phase and mobile phase of the chromatographic system respectively. *R* is the gas constant and *T* the temperature. The Henry coefficient was measured for all 20 AAs as shown in Figure 1a below. The binding free energy of all 20 AAs with the silica were evaluated using Umbrella Sampling (US) simulation and the binding affinity ($K_{calc}$ ) was calculated by integrating the free energy curve as follows^[48]^

(2) $$K_{calc}=C\int_{0}^{cutoff}dz\;exp(-\beta W_{calc}(z))$$

Here $W_{calc}\left(z\right)$ is the calculated free energy of binding for an AA to silica as a function of distance (z ) to the silica surface (see Experimental Section). *cutoff* is the distance up to which an AA is interacting with silica. A quantitative comparison of the calculated binding affinities and the experimental Henry coefficient is not possible because the constant C in Equation (2) cannot be determined. However, irrespective of this constant, the calculated binding affinity ($K_{calc}/C$ ) is proportional to the measured Henry coefficient (see Figure 1). The numerical values of the calculated free energy minima and the binding affinities of all the AAs are tabulated in Table S1 of the Supporting Information. As we observe from the Figure 1, the positively charged AAs arginine (R) and lysine (K) are the strongest binding AAs as revealed in both simulation and the experiment. These findings are in line with other experiments[^5^, ^6^, ^7^, ^32^] that indicate that the basic amino acids interact with silica at higher pH the most and the other AAs show low to no interaction at these conditions. The driving force for interaction of AAs with silica are the additional basic groups in R and K^[32]^ which provide strong electrostatic interactions with silica at high pH.^[37]^ It is important to note that in Figure 1 the experimental results are from the 10 mM TRIS pH 8 run and the simulation is in water. This is due to the problem, that in plain water the basic amino acids show high adsorption and thus no measurable retention time. This effect is mainly due to the competitive effect of TRIS on the AA adsorption and will be discussed in greater detail in the section below.

### 2.2 Influence of the Buffer

**Figure 2:**
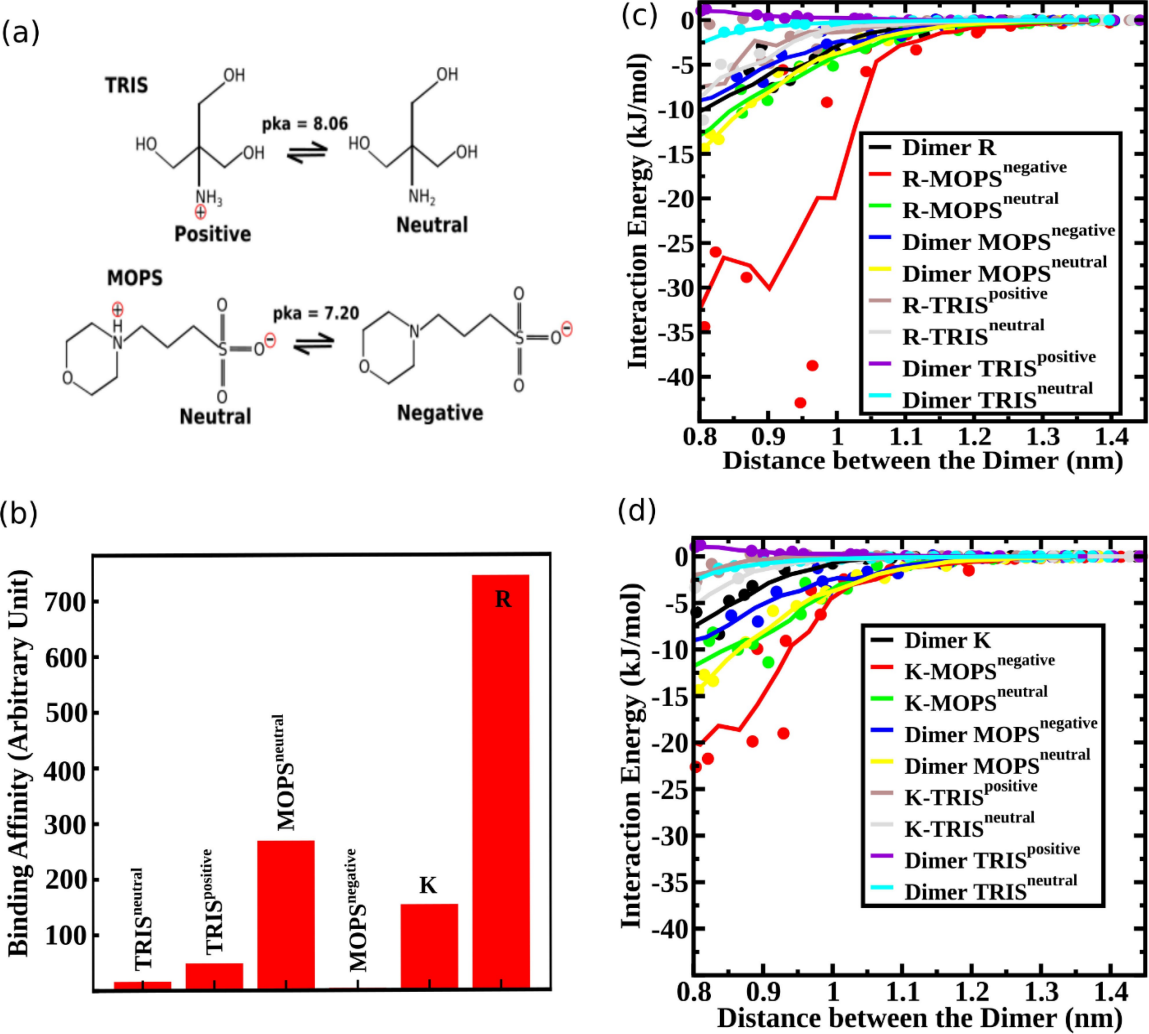
a) Molecular model of different protonation state of MOPS and TRIS buffer at different pH. TRIS has two protonation states with net charge +1e (TRIS^positive^) and 0 (TRIS^neutral^), while the protonation state of MOPS have net charge 0 (MOPS^neutral^) and −1e (MOPS^negative^). b) Binding affinities of the different buffer species to the silica. The binding affinities of K and R are also plotted for comparison. c) Interaction energy between the AA, R and different buffer species as a function of distance between them. d) Interaction energy between the AA, K and different buffer species as a function of distance between them. The interaction energy between the buffer species are plotted in both (c) and (d) for comparison. In both cases, there is a strong attraction between the R/K and the MOPS^negative^ species. (c‐d) The concentric circles in the figures indicate the actual calculated values while the solid lines are the running average over these data.

In the chromatography experiments, we used two different buffers: TRIS and MOPS. Depending on the pH of the solution, these buffers will have different protonation states as shown in Figure 2(a) below. TRIS has two protonation states with net charge +1e (TRIS^positive^) and 0 (TRIS^neutral^), while the protonation state of MOPS have net charge 0 (MOPS^neutral^) and −1e (MOPS^negative^). It has been previously reported that the buffer interacts with different oxide surfaces of titanium.^[11]^ Due to their charge the buffer species will also interact with silica and the AA, leading to competing interactions.[^11^, ^49^] Therefore, we evaluated the free energy of adsorption of the different buffer species to silica and also quantified the interaction between the buffer species and AAs. The binding affinity of the buffer species and the two strongly binding AAs (R and K) are shown in Figure 2b below. The corresponding free energy profile for the binding affinities are shown in Figure S1 and the Henry coefficient conversion for the experimental data in table S7 of the Supporting Information. As we observe from Figure 2b, the binding affinity of buffer species MOPS^neutral^ and TRIS^positive^ are quite comparable with amino acids K and R. Therefore, the buffer binding affinity cannot be ignored to determine the overall binding capacity of the AA in presence of the buffer. It's worth mentioning that although we have considered different protonation state of the buffer, we have only assumed the positively charged (protonated) species of R and K. However, this assumption is justified since the pKa values for corresponding amino group of R and K are 12.10 and 10.67 which is far above the pH range studied in this article. We further quantified the interaction between the amino acids (K and R) and the buffer species by calculating the interaction energy between them (see Experimental Section).

We can see from the Figure 2c, that the interaction energy is largest between R and MOPS^negative^ species. All other interaction energies are quite similar to the R−R dimer interaction. In case of K (Figure 2d) also, the strongest interaction was found to be between K and MOPS^negative^. The interaction energy at a specific distance between the two molecules are tabulated in Table S3 and S4 of the Supporting Information.

### 2.3 Binding of R and K to Silica in Presence of TRIS Buffer

Henry coefficient for the interaction of R and K were measured in presence of TRIS buffer for the pH range of 7.2 to 8.5. The binding affinity of both R and K increases with pH in presence of TRIS buffer. This effect is expected due to the increasing negative charge of silica surface with increasing pH.[^32^, ^50^] Nevertheless, TRIS buffer has a strong impact on the binding of AA because we were not able to gain Henry coefficients in plain water due to strong interactions of K and R with the silica surface. This can be explained by the higher ionic strength of the solution through the buffer and thus a competitive absorption of TRIS and the AA.^[50]^ Although higher pH leads to higher surface charge of silica the increase of the Henry coefficient is not comparable to adsorption experiments in the same pH range showing a more linear like increase.[^33^, ^50^]

To understand this pH dependent interaction of R and K in presence of buffer, we set‐up a multiscale modelling framework for adsorption. The multiscale modelling consists of calculations of energetic parameters of binding from the MD simulation (see Experimental Section) and further use of these parameters in two mechanistically different multicomponent Langmuir adsorption models. The models provide the fraction of R/K bound to silica which is again proportional to the measured Henry coefficient. Depending on the interaction strength between the AA and the buffer species in an adsorbed state, we invoke one of the two different kinds of Langmuir models^[51]^ as shown schematically in Figure 4 below. As the name suggests, in the non‐cooperative model (Figure 4a) the interaction between the adsorbates (A and B) are neglected while the cooperative model (Figure 4b) is formulated assuming an interaction between the adsorbates. The black semicircles are the adsorption sites (silica in our case) which can either accommodate one (non‐cooperative model) or two (cooperative model) adsorbates (A and B). In an equilibrium situation, the adsorptive molecules continuously adsorb and desorb (see Supporting Information). The type of multicomponent cooperative adsorption model we consider here was first derived by Moreau et al.^[52]^ and therefore also known as the Moreau model in the literature. Although, Langmuir model was originally developed to study adsorption from the gas phase, the model is much more general and appears in variety of other physical situations (e. g. ligand binding to protein) described by a simple combination reaction where loss of mass action is valid in equilibrium.^[51]^ Therefore, use of Langmuir model to describe adsorption in Liquid‐Solid interface is fully justified. Esposito et al.^[53]^ fitted their experimental adsorption isotherm with this kind of multicomponent Langmuir model to study the bio‐separation of metal ions. The pH dependence of the isotherms was captured in the ratio of the different protonated and unprotonated metal ions. Xiao et al.^[54]^ measured the adsorption isotherm of several organic acids and bases on graphite and fitted the isotherm with the multicomponent Langmuir model. The pH dependence was captured by the protonation and deprotonation of the organic acid and bases similar to the work by Esposito et al.^[53]^

**Figure 3:**
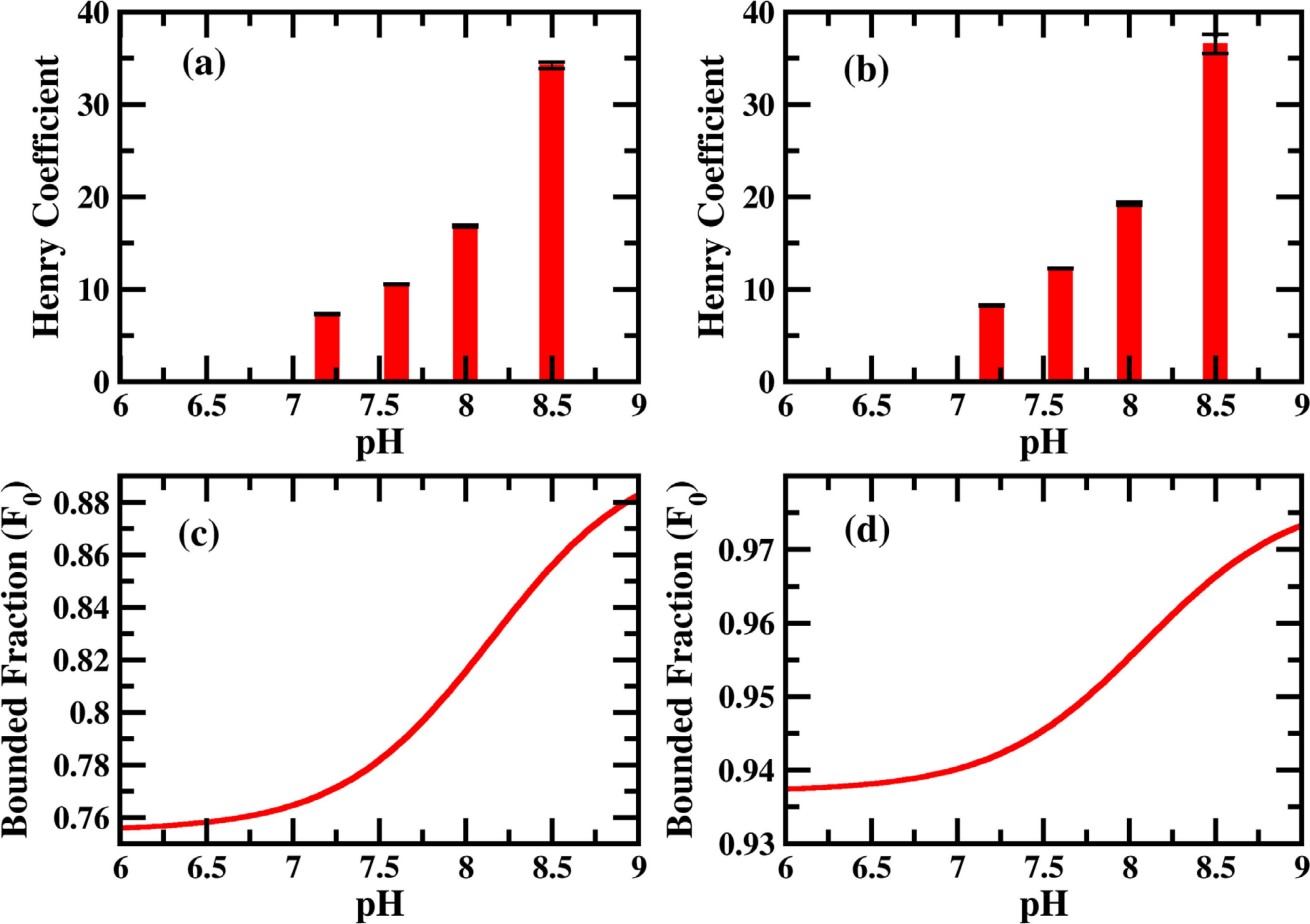
Measured Henry coefficient of K (a) and R (b) as a function of pH in presence of TRIS buffer. pH values are quoted on top of the bars. Fraction of K (c) and R (d) bound to silica as a function of pH as calculated using multiscale modelling. The experimental Henry coefficient and calculated bounded fraction show qualitatively similar behaviors.

**Figure 4:**
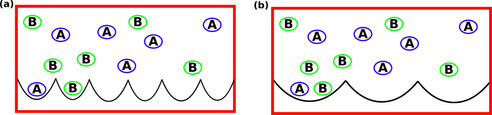
a) Schematic diagram illustrating non‐cooperative competitive Langmuir adsorption model of two different species A and B. The black semi circles represent the adsorption sites which can accommodate only one molecule. There is no interaction between A and B. b) Schematic diagram illustrating a cooperative adsorption model of two different species A and B. The black semi circles now represent adsorption sites which can accommodate up to two molecules. There is an interaction between the molecular species when adsorbed in a same adsorption site.

Since, there is no strong interaction between different molecular species (see Figure 2c,d) and Table S3 and S4 in the supporting information) in presence of TRIS buffer, we will invoke the non‐cooperative (Figure 4a) competitive Langmuir model to understand the pH dependent binding as described in the Experimental Section in detail. We calculate the fraction of bound AA for different pH (Figure 3c,d) and found qualitatively same behavior with the experiment. The increase of binding fraction of K/R with the pH can be simply understood as follows. When the pH of the solution is low (<8), there will be more TRIS^positive^ species in the solution than the TRIS^neutral^ (see Figure 2a). Therefore, the effect is simply a competitive effect between TRIS and the AA for the negatively charged silica surface. With the increase in pH, the TRIS^positive^ species will deprotonate giving rise to more TRIS^neutral^ species. Therefore, as pH increases, K/R has to compete with TRIS^neutral^ for the binding sites while for low pH the competition for the binding sites will be between the K/R and TRIS^positive^ which has a much higher binding affinity (to silica) in comparison to TRIS^neutral^. As a result, the binding fraction of K/R increases significantly when the pH is higher than the pka of the TRIS buffer.

### 2.4 Binding of R and K to Silica in Presence of MOPS Buffer

**Figure 5:**
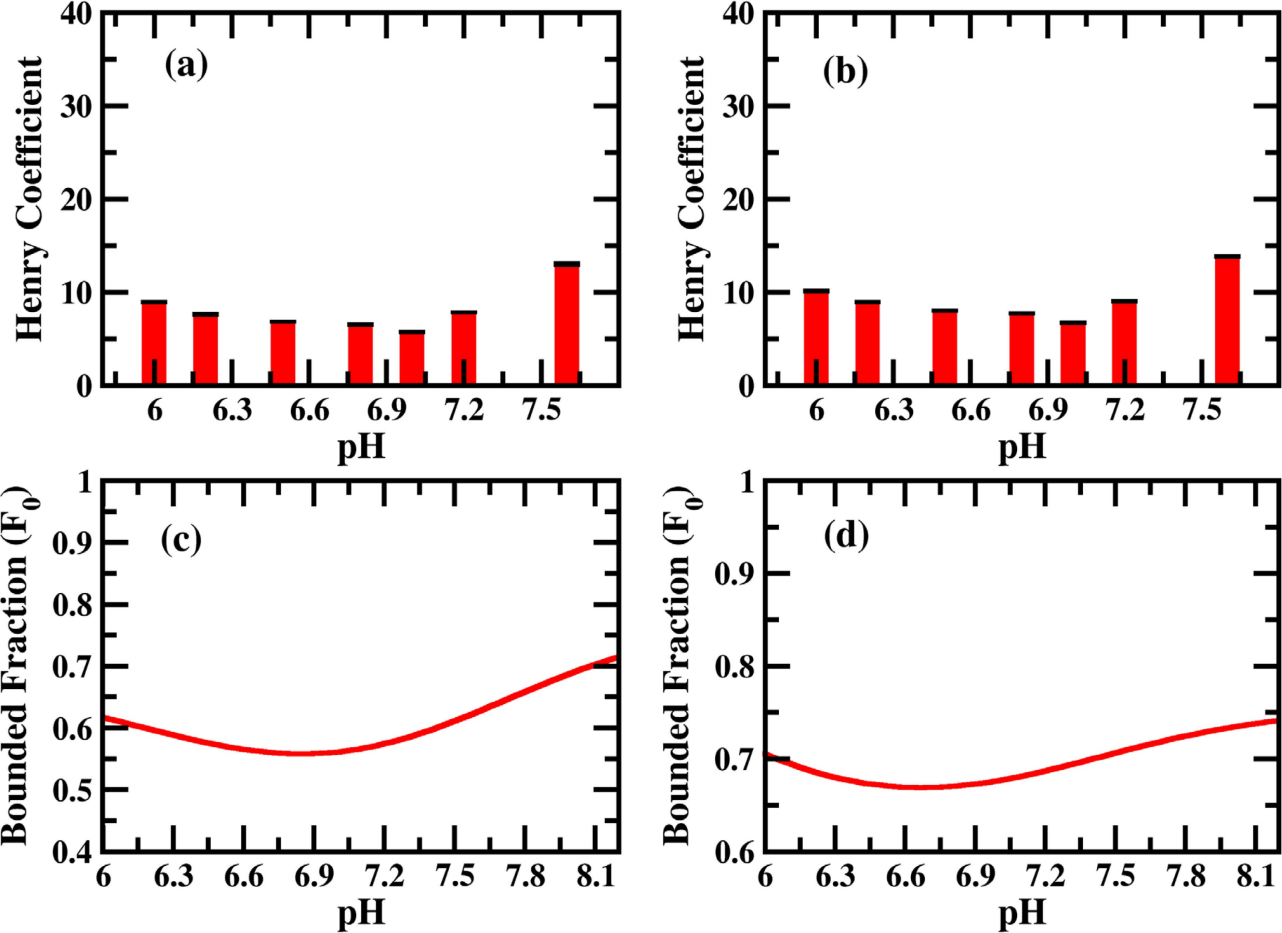
a)/b) Experimentally measured Henry coefficients of K/R as a function of pH in presence of MOPS buffer. pH values are quoted on top of the bars. c)/d) Fraction of K/R bound to silica calculated using multiscale modeling.

Retention factors for the binding of R and K were again measured in presence of MOPS buffer for the pH range of 6 to 7.6 and converted in Henry coefficient (see table S8 of Supporting Information). As shown in Figure 5a,b below, for a pH range 6 to 7.2 the interaction of both the AAs slowly decreases and at a pH of 7.6 we see a sudden increase in interaction. Previous experiments always indicated higher adsorption capacities with increasing pH.[^32^, ^33^, ^50^] To understand the experimental binding behavior, we again use our multiscale modelling framework as described in the previous section. Since the deprotonated species of the MOPS buffer (MOPS^negative^) has a net attractive interaction (see Figure 2c,d)) with the R/K species, the binding will be described by the cooperative Langmuir adsorption model (also known as Moreau model^[52]^) rather than by the non‐cooperative one (see Figure 4b). Gritti et al.^[55]^ measured the adsorption isotherm of various alcohol with porous silica and fitted the isotherm with non‐cooperative and cooperative Langmuir model (also known as Moreau model) depending on whether the alcohol solution is buffered or not. Neither the interaction between the solid phase and the buffer nor the interaction between the buffer and the alcohol was considered in their models. Figure 5a,b show the experimentally measured Henry coefficients for K/R, while Figure 5c,d shows the calculated fraction of bound K/R (see Experimental Section). We observe a good qualitative match between the experiment and the modelling for this scenario.

The physical origin of the binding behavior of K/R with respect to pH can be understood as follows. Since MOPS buffer has a pKa of 7.2, for low pH (<7.2), the solution in the chromatographic column will be populated by MOPS^neutral^ species, while for high pH (>7.2) most of the MOPS^neutral^ species will be deprotonated and as a result there will be more MOPS^negative^ species in the solution (see Figure 2a). If there is no interaction between the K/R and MOPS, for low pH, K/R has to compete with MOPS^neutral^ while for high pH, it has to compete with MOPS^negative^ species for silica binding sites. Since MOPS^neutral^ has a much higher binding affinity than the MOPS^negative^, the K/R binding fraction will increase with increasing pH. However, the situation is different when there is a moderate attraction between K/R and MOPS^negative^. Since MOPS^negative^ has a pretty low binding affinity, it cannot compete with K/R for binding sites on its own. However, due to the strong attraction of the K/R with MOPS^negative^, when a K/R binds to silica, it sometimes accompanies a MOPS^negative^ with it and as a result the binding sites are occupied by MOPS^negative^ also. Therefore, the overall binding of K/R decreases with increasing concentration of MOPS^negative^ and seems to be more relevant than the increasing negative charge of the silica surface with increasing pH. However, as the pH increases further, the concentration of MOPS^negative^ increases together with the negative surface charge of the silica.^[32]^ K/R cannot interact with additional MOPS^negative^ molecules to form complexes. Due to a decrease in MOPS^neutral^ concentration and increased negative charge on the surface more silica binding sites are available for K/R, resulting in an increased overall binding in the end.

## 3 Conclusions

To summarize, we have measured the binding affinity of all 20 AAs with silica using zonal elution chromatography. We could show the capability of chromatography for studying interactions between single AAs and silica surfaces under different conditions in aqueous systems. Furthermore, chromatography has the advantage of real time monitoring and the possibility to have an automated high throughput system, which leads to a lot of data points with little effort. Among the 20 AAs, the positively charged AAs R and K were found to have highest affinity towards silica, which was validated by calculation of binding free energy using US simulation. The binding behavior of R and K was further studied in presence of different buffers and was found out be strongly dependent on the choice of buffer which is never accounted in biotechnology experiments. When TRIS was used as buffer the binding affinity of R/K increases with pH (7.3 to 8.5) while in case of MOPS the affinity first decreases with pH (6.0 to 7.0) and with further increase of pH (>7) the affinity again goes up. In addition, with its conventional role, the buffer can be used to tune the AA‐silica interaction increasing the efficiency of AA separation, by a significant amount. We also present a multiscale modelling framework to understand the binding of AA in presence of buffer. The multiscale modelling consists of calculations of energetic parameters of binding from the MD simulation and further use of these parameters in mechanistically different multicomponent Langmuir models. In a very recent work, similar multiscale modelling approach involving MD simulation and the non‐cooperative Langmuir model was adopted by Angelis et al.^[56]^ to predict the adsorption of surfactant to the alumina. In this work, we extend the Langmuir model to account for multiple interacting species which is relevant in variety of physical situations including ours. The multiscale‐modeling framework can be used to screen the suitable buffer prior to the experiment, which is often expensive, and time consuming to perform. Our model helps to predict the relative interactions strength between different components in a bio molecular mixture (AA, peptides, proteins etc.) appears in variety of physical situations like chromatographic purification. We hope to extend the cooperative adsorption model in the future to describe the incorporation of peptides and proteins in mesoporous silica materials as well.

## Experimental Section

**Adsorbent and AAs**: The silica used for the experiments was Silica Gel 60 from AppliChem, Germany. The porous silica had a particle size of 40 to 60 μm. The pore diameter was 55 to 65 Å and the pore volume 0.7 to 0.8 mL g^−1^. The surface area was given with 450 to 550 m^2^ g^−1^. TRIS was purchased from VWR, Germany. MOPS was purchased from Carl Roth, Germany. All AAs were purchased as L‐stereoisomer. Most AAs were purchased in research grade from SERVA, Germany. Arginine, histidine and proline (Cellpure≥98 %) were purchased from Carl Roth, Germany. Cysteine, lysine and phenylalanine were purchased with a purity ≥98 % from Sigma‐Aldrich, Germany. For the column, a column blank kit (Supelco) with L×ID 25 cm×4.6 mm from Sigma‐Aldrich was purchased and shortened to a length of 3.3 cm resulting in a volume of 0.55 mL. The buffers were prepared in DI water. The AAs were also prepared in DI water with concentrations between 1 and 50 mM (see Table S5 of the Supporting Information). All buffers were degassed and filtered through a 0.2 μm cellulose‐acetate‐filter from Labsolute, Germany. The AAs were also filtered with 0.2 μm cellulose‐acetate syringe filters from Macherey‐Nagel, Germany.

**Henry coefficients**: The chromatographic column was operated on an Agilent 1100 HPLC system with an UV/Vis detector. AAs were measured at 210 nm, aromatic AAs at 280 nm additionally. The flow rate was ∼12 cm min^−1^ for every run and the injection volume for every AA was 20 μL. Every AA was measured at least three times per experiment in random sequences. The Henry coefficients H was determined with H=k’/φ. Where k’ is the retention factor of the AA and φ is the phase ratio of the column. The retention factor is calculated as k’=(t_R_−t_0_)/t_0_. Here t_R_ stands for the retention time of the AA and t_0_ for the retention time of a non‐interacting tracer in this case 1 g L^−1^ uracil. The phase ratio of the column is calculated with φ=(1−ϵ^t^)/ϵ^t^. Here ϵ^t^ is the total porosity of the column calculated with the flow rate $\dot{V}$ =2 mL min^−1^:ϵ^t^=(t_0_ $\;\dot{V)}$ /V_column_.

**The Langmuir adsorption model for two different non‐interacting adsorbates (non‐cooperative Langmuir model)**: Consider two adsorbates A and B (See Figure 4a) with binding affinity$\;{K_{A}}^{'}$ and ${K_{B}}^{'}$ with the adsorbent having a total Γ number of adsorption sites. The average number of A molecule bound ($N_{A}$ ) is given by^[51]^

(3) $$<N_{A}>\frac{}{\Gamma }=F_{A}=\frac{{K_{A}}^{{}^{'}}\theta_{A}}{1+\theta_{A}{K^{{}^{'}}}_{A}+\theta_{B}{K^{{}^{'}}}_{B}}$$

See the Supporting Information for more details.

**The Langmuir adsorption model for two different interacting adsorbates (cooperative Langmuir model)**: Consider two adsorbates A and B with binding affinity$\;{K_{A}}^{'}$ and ${K_{B}}^{'}$ with the adsorbent having a total Γ number of adsorption sites (see Figure 4b). $U_{AA}$ is the interaction energy between two A molecules when both of these two are adsorbed on a single adsorption site. $U_{BB}$ is the corresponding interaction energy for the B molecules and $U_{AB}$ is the interaction energy between A and B in case the adsorption site is occupied by one A and one B molecule respectively. In equilibrium, the average number of A molecule bound ($N_{A}$ ) is given by[^51^, ^52^]

(4) $$<N_{A}>/\Gamma =F_{A}=\;\frac{2{K_{A}}^{'}\theta_{A}+2{\theta_{A}}^{2}{{K^{'}}_{A}}^{2}e^{-\beta U_{AA}}+{2\theta }_{A}{K^{'}}_{A}\theta_{B}{K^{'}}_{B}e^{-\beta U_{AB}}}{\begin{array}{c} 1+2\theta_{A}{K^{{}^{'}}}_{A}+{\theta_{A}}^{2}{{K^{{}^{'}}}_{A}}^{2}e^{-\beta U_{AA}}+\\ {{2\theta_{B}{K^{{}^{'}}}_{B}+\theta }_{B}}^{2}{{K^{{}^{'}}}_{B}}^{2}e^{-\beta U_{BB}}+{2\theta }_{A}{{K^{{}^{'}}}_{A}\theta }_{B}{K^{{}^{'}}}_{B}e^{-\beta U_{AB}} \end{array}}$$

The complete derivation of Equation (4) above is presented in the Supporting Information.

**Figure 6:**
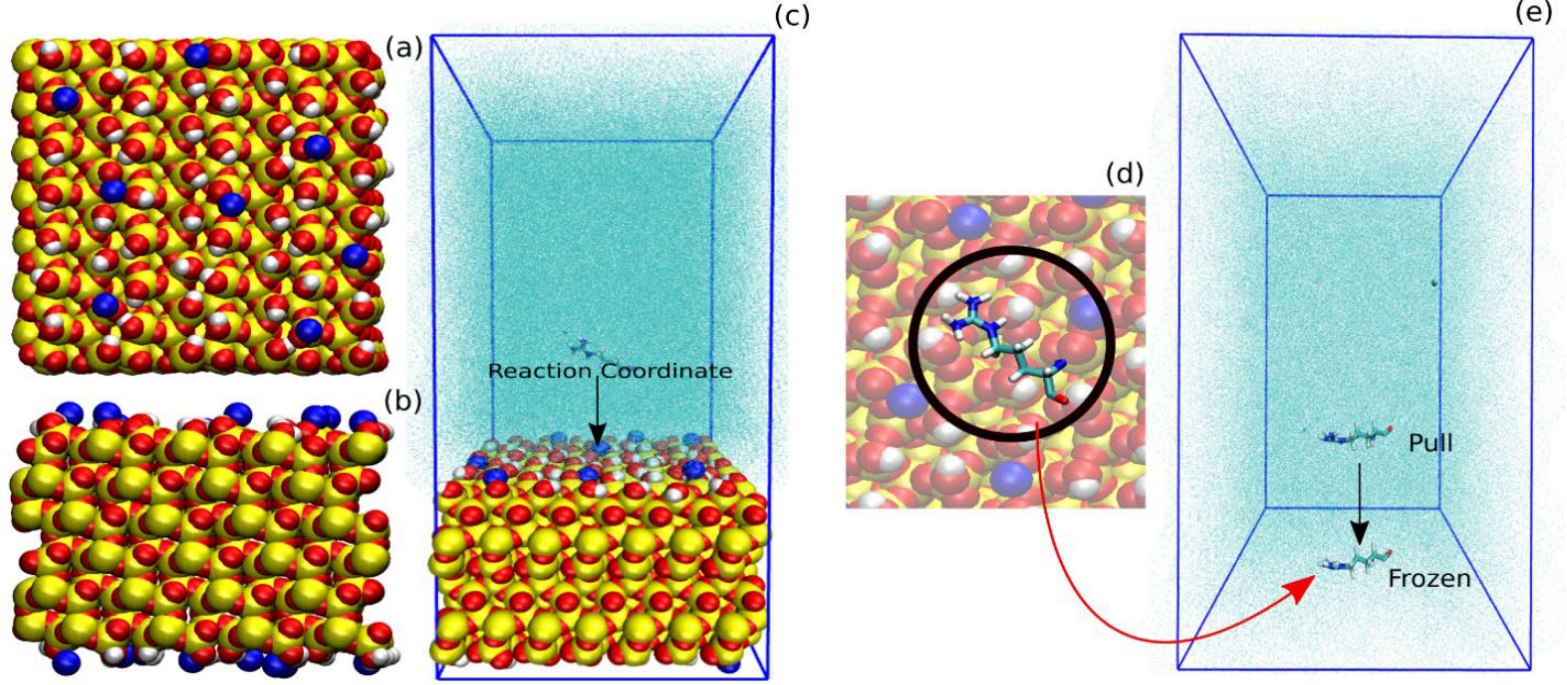
Atomistic model of the silica used in the MD simulation: a) Top and b) side view. Silicon atoms are represented in yellow, oxygen atoms in red and hydrogens in white. Na^+^ ions are shown in blue. c) Snapshot of the initial system prepared for MD simulation with silica in the end and the AA in the middle of the simulation box. The surrounding water medium is not shown in full atomistic details but as collection of cyan dots (“solvent” representation in VMD^[63]^) for clarity. The free energy of binding was computed as a function of distance between the AA and Silica (Reaction Coordinate). d) Equilibrated snapshot of the AA adsorbed on silica. e) Initial snapshot of a system prepared to calculate the interaction energy between the adsorbed AA and another molecule. The AA was kept frozen to its adsorbed geometry while another molecule is pulled towards it and the interaction energy is measured.

**Binding affinity of the AAs and the buffers to the silica surface**: To estimate the binding affinity of the different molecular species with the silica surface we calculate the potential of mean force (PMF) (between the molecule and silica) of binding using umbrella sampling (US) simulation. The atomistic model (Figure 6a,b) of the porous silica surface for the MD simulation was chosen from the database provided by Emami et al.^[21]^ From the database we chose a $Q^{3}$ silica surface model (33.6 Å×34.9 Å) containing 4.7 silanol groups per nm^2^ of the surface of which 14 % are deprotonated corresponding to a pH of 7.4. The model for the AAs were built using Ambertools^[57]^ program. A simulation box with the silica in one end and AA in the middle was prepared. The full system was then solvated in TIP3P water^[58]^ (Figure 6c). A sufficient number of Na^+^ and Cl^−^ counter ions were added to achieve overall charge neutrality of the system. The force field for the silica surface were taken from Emami et al.^[21]^ while AMBER99SB‐ILDN^[59]^ force field was used for AAs including the solvents. The system (∼10,000 atoms) was first energy minimized and then MD simulation in isothermal ensemble (NVT) was performed to equilibrate the system. The silica surface was kept frozen during the simulation and periodic boundary condition was imposed in all three directions. The x‐ and y‐dimensions of the box were kept equal to the x‐ and y‐dimensions of the silica surface, and the atoms located at the edge of the silica patch were connected through bonds via the periodic boundary condition to avoid boundary effects. A series of short NVT simulations with varying z dimension of the box were performed thereafter to achieve the correct density of the water in the bulk. We used Nose‐Hoover thermostat^[60]^ to maintain the system temperature at 300 K. The system with correct water density was further used for US simulations. All the simulations were performed using GROMACS^[61]^ simulation package. We further proceed to calculate PMF of AA with the silica surface using US simulation with the distance between the silica surface and the center of mass of the AA as reaction coordinate (Figure 6c). To generate the configuration for US run, the AA was pulled towards the silica surface and the overall system is equilibrated again when the AA is adsorbed on top of the silica surface. The AA is then pulled off from the silica surface and the system configuration is saved at a regular distance (between the AA and the silica surface) interval for the umbrella sampling run. We used a spring constant of 1000 kJ mol/nm^2^ and the pull rate 0.01 nm/ps for the pulling simulations. The umbrella sampling simulation were further performed with these configurations with the strength of the umbrella potential 1000 kJ mol/nm^2^. Each umbrella sampling windows were first equilibrated for 4 ns and then from another 10 ns run we save the histograms for PMF generation. The PMF curves were calculated using the Weighted Histogram Analysis Method (WHAM)^[62]^ also implemented in GROMACS. The obtained histograms and the PMF profile for a specific case are shown in Figure S5 of the Supporting Information. In this article, we use the term free energy profile as alternative to the PMF profile both having same meaning.

**Interaction energy between R/K and the buffer species**: To measure the interaction energy of R/K with another R/K or the different buffer species, we chose the equilibrated geometry of R/K as it adsorbed on the silica (Figure 6d). The R/K was kept frozen in that geometry while other molecule (another R/K or buffer species) was pulled towards it (Figure 6e) and the interaction energy was measured. The interaction energy between different buffer‐species was also measured the same way.

**The multiscale modelling (binding of K/R in presence of TRIS)**: There are 3 different molecular species present in the chromatography column in this case: K/R and two buffer species TRIS^neutral^ and TRIS^positive^ (see Figure 2a). The concentration of K/R in the chromatographic column is $X_{0}$ and the concentration of the TRIS^neutral^ and TRIS^positive^ buffer species are $X_{1}$ and $X_{2}$ . If the total concentration of the buffer species is $C_{B}$ and pka is the buffer pKa value, then

(5) $$X_{1}+X_{2}=C_{B}\;\mathrm{and}\;X_{1}/X_{2}=10^{ph-pka}$$

For a given value of $C_{B}$ , Equation (5) can be solved for $X_{1}$ and $X_{2}$ at a particular pH.

According to Equation (3), the total fraction of R/K bound $F_{0}$ is given by,

(6) $$F_{0}=\left(\frac{X_{0}K_{0}}{1+X_{0}K_{0}+X_{1}K_{1}+X_{2}K_{2}}\right)$$

Here $K_{0},\;K_{1}$ and $K_{2}$ are the binding affinity for AA, TRIS^neutral^ and TRIS^positive^ respectively. Please note that the binding affinities in Equation (6) and the ones calculated in the simulation (Equation 2) are not quantitatively same but proportional to each other. The use of the binding affinity from the simulation in the Equation (6) is still justified, if one is not looking for quantitative prediction but the qualitative behavior. In all our calculation, the concentration of both buffer ($C_{B}$ ) and AA ($X_{0}$ ) was assumed to be 1 (arbitrary unit).

**The multiscale modelling (binding of K/R in presence of MOPS)**: As before, there are 3 different molecular species present in the chromatography column: K/R and two buffer species MOPS^neutral^ and MOPS^negative^ (see Figure 2a). The concentration of K/R in the chromatographic column is $X_{0}$ and the concentration of the MOPS^negative^ and MOPS^neutral^ buffer species are $X_{1}$ and $X_{2}$ . We can write equation similar to Equation (5) as

(7) $$X_{1}+X_{2}=C_{B}\;\mathrm{and}\;X_{1}/X_{2}=10^{ph-pka}$$

Now, the total fraction of K/R bound to silica is given by

(8) $$F_{0}=1/2\left(F_{01}+F_{02}\right)$$

Here, $F_{01}$ is the fraction of K/R bound to silica due to cooperative adsorption between K/R and MOPS^negative^. $F_{02}$ is the corresponding fraction when K/R and MOPS^neutral^ are considered. According to Equation (4), we can write

(9) $$F_{01}=\;\frac{2X_{0}K_{0}+2{X_{0}}^{2}{K_{0}}^{2}exp\left(-\beta U_{00}\right)+X_{0}K_{0}X_{1}K_{1}exp\left(-\beta U_{01}\right)}{\begin{array}{c} 1+2X_{0}K_{0}+{X_{0}}^{2}{K_{0}}^{2}\exp\left(-\beta U_{00}\right)+2X_{1}K_{1}+\\ {X_{1}}^{2}{K_{1}}^{2}exp\left(-\beta U_{11}\right)+X_{0}K_{0}X_{1}K_{1}exp\left(-\beta U_{01}\right) \end{array}}$$

(10) $$F_{02}=\;\frac{2X_{0}K_{0}+2{X_{0}}^{2}{K_{0}}^{2}exp\left(-\beta U_{00}\right)+X_{0}K_{0}X_{2}K_{2}exp\left(-\beta U_{02}\right)}{\begin{array}{c} 1+2X_{0}K_{0}+{X_{0}}^{2}{K_{0}}^{2}\exp\left(-\beta U_{00}\right)+2X_{2}K_{2}+\\ {X_{2}}^{2}{K_{2}}^{2}exp\left(-\beta U_{22}\right)+X_{0}K_{0}P_{2}K_{2}exp\left(-\beta U_{02}\right) \end{array}}$$

The parameter $U{}^{'}s$ are the interaction between different species and $K{}^{'}s$ are the binding affinity. Among the different intermolecular interaction energies in Equations (9) and (10), only the interaction energy between the R/K and MOPS^negative^ ($U_{01}$ ) is significant (see Figure 2c,d). Therefore we keep all other intermolecular interaction energies ($U_{00},\;U_{02},U_{11},U_{22}$ ) zero except$\;U_{01}$ . In case of binding of R we use a value of $U_{01}$ to be −20 kJ/mol while in case of K the value is −12 kJ/mol (see Table S3 and S4 of the Supporting Information) and get the binding behavior as shown in Figure 5c,d. In all our calculation, the concentration of both buffer ($C_{B}$ ) and AA ($X_{0}$ ) was assumed to be 1 (arbitrary unit). Please note that the interaction energies are calculated at a distance of 8 Å between the species (see Table S3 and S4 of the Supporting Information) which may not be the case in reality. Therefore, we calculate the binding fraction of R/K with pH for different value of $U_{01}$ . We observe ( see Figure S6 of Supporting Information) that in case of R, an attractive interaction (between the R and MOPS^negative^) of magnitude >11 kJ/mol is required to qualitatively reproduce the experimental behavior while in case of K the respective interaction energy is 7 kJ/mol. It is evident from the Figure 2c,d (and Tables S3 and S4 of the Supporting Information) that R and MOPS^negative^ have higher attractive interaction than K and MOPS^negative^.

## Conflict of interest

The authors declare no conflict of interest.

## Supporting information

As a service to our authors and readers, this journal provides supporting information supplied by the authors. Such materials are peer reviewed and may be re‐organized for online delivery, but are not copy‐edited or typeset. Technical support issues arising from supporting information (other than missing files) should be addressed to the authors.

Supplementary
