# Supplementary material for: Buffer Influence on the Amino Acid Silica Interaction
Source: Chemphyschem. 2020 Sep 23;21(20):2347–56. doi: 10.1002/cphc.202000572 (PMC7702087; doi:10.1002/cphc.202000572)
Supplement: Supplementary file 1 — Supplementary [file CPHC-21-2347-s001.pdf]

ChemPhysChem

Supporting Information

Buffer Influence on the Amino Acid Silica Interaction

Saientan Bag+, Stefan Rauwolf+, Mikhail Suyetin, Sebastian P. Schwaminger, Wolfgang Wenzel,* and Sonja Berensmeier*

Supporting Information

Table S1. Minima of the PMF profile and the binding affinity of all 20 amino acids with the silica calculated using umbrella sampling simulation.

Amino acid	Amino Acid 1 letter code	PMF Minima (kJoule/mol)	Binding Affinity [Kcalc/C] (Arbitrary Unit)
Glycine	G	-8.3	3
Alanine	A	-9.2	5
Valine	V	-12.5	18
Leucine	L	-14.0	21
Isoleucine	I	-14.3	34
Proline	P	-11.5	10
Methionine	M	-14.2	24
Cysteine	C	-9.7	5
Asparagine	N	-11.8	11
Glutamine	Q	-13.6	20
Aspartic acid	D	-9.2	0.8
Glutamic acid	E	-5.7	1
Serine	S	-9.9	5
Threonine	T	-9.8	6
Tyrosine	Y	-15.4	46
Phenylalanine	F	-13.6	19
Tryptophan	W	-15.4	43
Histidine	H	-13.0	20
Lysine	K	-18.6	153
Arginine	R	-23.3	745

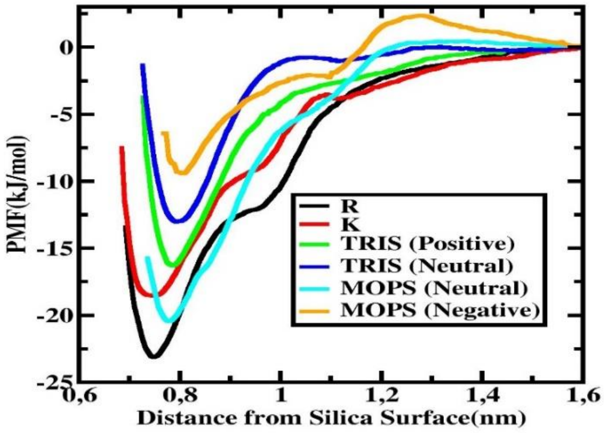

Figure S1. PMF profile of R and K and the buffer species.

Table S2. Minima of the PMF profile and the binding affinity of different buffer species. The numbers for R and K are also quoted for comparison.

Molecular Species	PMF minima (kJoule/mol)	Binding Affinity (K_{calc}/C) (Arbitrary Unit)
TRIS (neutral)	-13.0	16
TRIS (positive)	-16.4	49
MOPS (neutral)	-20.5	269
MOPS(Negative)	-9.5	4
K	-18.6	154
R	-23.3	745

Table S3. Interaction energy between two molecular species for R.

Species 1	Species 2	Interaction Energy (kJ/mol)*	Interaction Energy with respect to Arginine dimer (kJ/mol)*
Arginine	Arginine	-10	0
Arginine	MOPS(Negative)	-30	-20
Arginine	MOPS(Neutral)	-13	-3
Arginine	TRIS(Positive)	-7	3
Arginine	TRIS(Neutral)	-7	3
MOPS(Negative)	MOPS(Negative)	-10	0
MOPS(Neutral)	MOPS(Neutral)	-13	-3
TRIS(Positive)	TRIS(Positive)	1	11
TRIS(Neutral)	TRIS(Neutral)	-3	7

*interaction energies are reported at a separation of 8Å between the species

Table S4. Interaction energy between two molecular species for K.

Species 1	Species 2	Interaction Energy (kJ/mol)*	Interaction Energy with respect to Lysine dimer (kJ/mol)*
Lysine	Lysine	-8	0
Lysine	MOPS(Negative)	-20	-12
Lysine	MOPS(Neutral)	-12	-4
Lysine	TRIS(Positive)	-3	5
Lysine	TRIS(Neutral)	-5	5
MOPS(Negative)	MOPS(Negative)	-10	-2
MOPS(Neutral)	MOPS(Neutral)	-13	-5
TRIS(Positive)	TRIS(Positive)	1	9
TRIS(Neutral)	TRIS(Neutral)	-3	5

*interaction energies are reported at a separation of 8Å between the species

Derivation of Cooperative Langmuir Model

Let's assume there are two different adsorbate species A and B. The species are assumed to be in the ideal gas phase unless these are adsorbed on the surface adsorption sites (shown as the black parabola in the schematic in Fig. S2) of the adsorbent. Each adsorption site can accommodate a maximum of two molecules where the adsorption phenomenon is governed by adsorbate-adsorbent interaction as well as adsorbate-adsorbate interaction.

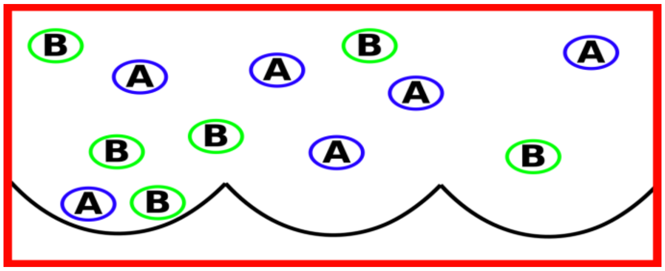

Figure S2. Schematic diagram representing a cooperative adsorption model of two different species A and B. The black semi circles are the adsorption sites, which can accommodate up to two molecules.

To formulate the statistical mechanics of this adsorption processes we first enumerate the different number of microstates for a single adsorption site (see the schematic in Fig.S3) as follows:

1. Adsorption site is empty.
2. Occupied by one A molecules and an empty spot.
3. Occupied by two A molecules.
4. Occupied by one B molecules and an empty spot.
5. Occupied by two B molecules.

6. Occupied by one A and one B molecules.

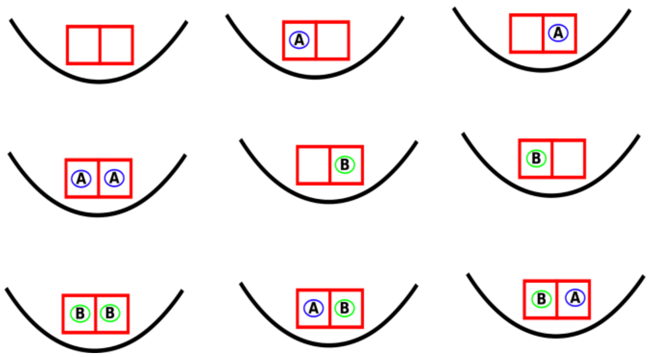

Figure S3. Different microstates for a single adsorption site (capable of holding two adsorbate molecules) in the presence of two adsorbates A and B.

U_A and U_B are the potential energy of the molecule A and B due to interaction with the adsorbent upon adsorption. U_{AA} is the interaction energy between two A molecules when both of these two are adsorbed on a single adsorption site. U_{BB} is the corresponding interaction energy for the B molecules and U_{AB} is the interaction energy between A and B in case the adsorption site is occupied by one A and one B molecule respectively.

The grand canonical partition function for the single adsorption site is then given by

$$\xi = 1 + 2{\Lambda_A}^{-3}Ve^{-\beta U_A}e^{\beta\mu_A} + 2{\Lambda_B}^{-3}Ve^{-\beta U_B}e^{\beta\mu_B} + {\Lambda_A}^{-6}V^2e^{-\beta(2U_A+U_{AA})}e^{2\beta\mu_A} + {\Lambda_B}^{-6}V^2e^{-\beta(2U_B+U_{BB})}e^{2\beta\mu_B} + 2{\Lambda_A}^{-3}V^2{\Lambda_B}^{-3}V^2e^{-\beta(U_A+U_B+U_{AB})}e^{\beta\mu_A}e^{\beta\mu_B} \quad (1)$$

Λ_A and Λ_B are the thermal de Broglie wavelength of the adsorbate molecule A and B respectively. V is the free volume offered by half of the adsorption site. Here, the terms ${\Lambda_A}^{-3}V$ and ${\Lambda_B}^{-3}V$ come from the configurational part of the partition function. μ_A and μ_B are the chemical potential of the molecular A and B respectively. Here, $\beta = 1/K_BT$. K_B is the Boltzmann constant and T is the temperature.

Now, if there are Γ independent adsorption sites, the total grand canonical partition function of the system is

$$\Xi = \xi^{\Gamma} \quad (2)$$

The expectation value of the number of adsorbed molecule A is then given by

$$< N_A >= \left(\frac{\partial log\Xi}{\partial(\beta\mu_A)}\right) \quad (3)$$

$$= \Gamma \frac{2{\Lambda_A}^{-3}Ve^{-\beta U_A}e^{\beta\mu_A} + 2{\Lambda_A}^{-6}V^2e^{-\beta(2U_A+U_{AA})}e^{2\beta\mu_A} + 2{\Lambda_A}^{-3}V^2{\Lambda_B}^{-3}V^2e^{-\beta(U_A+U_B+U_{AB})}e^{\beta\mu_A}e^{\beta\mu_B}}{1 + 2{\Lambda_A}^{-3}Ve^{-\beta U_A}e^{\beta\mu_A} + 2{\Lambda_B}^{-3}Ve^{-\beta U_B}e^{\beta\mu_B} + {\Lambda_A}^{-6}V^2e^{-\beta(2U_A+U_{AA})}e^{2\beta\mu_A} + {\Lambda_B}^{-6}V^2e^{-\beta(2U_B+U_{BB})}e^{2\beta\mu_B} + 2{\Lambda_A}^{-3}V^2{\Lambda_B}^{-3}V^2e^{-\beta(U_A+U_B+U_{AB})}e^{\beta\mu_A}e^{\beta\mu_B}}$$

Now, at equilibrium, the chemical potential of the molecular species A and B are related to their partial pressure according to following thermodynamic condition

$$\mu_A = \frac{1}{\beta} log({\Lambda_A}^3 \beta P_A); \quad \mu_B = \frac{1}{\beta} log({\Lambda_B}^3 \beta P_B) \tag{4}$$

And the Langmuir Binding Constant for the molecular species A and B defined as

$$K_A = \beta V e^{(-\beta U_A)}; K_B = \beta V e^{(-\beta U_B)} \tag{5}$$

Using equations (4) and (5), equation (3) can be rewritten in the following form

$$< N_A >= \Gamma \frac{2P_A K_A + 2{P_A}^2 {K_A}^2 e^{-\beta U_{AA}} + 2P_A K_A P_B K_B e^{-\beta U_{AB}}}{1 + 2P_A K_A + {P_A}^2 {K_A}^2 e^{-\beta U_{AA}} + 2P_B K_B + {P_B}^2 {K_B}^2 e^{-\beta U_{BB}} + 2P_A K_A P_B K_B e^{-\beta U_{AB}}} \tag{6}$$

Similarly, we can write another equation for the expectation value of adsorbed molecule B. The equation (6) above is written in terms of the partial pressure of the adsorbates. Alternatively, it can be rewritten in terms of the equilibrium concentration of the adsorbates. If $\theta_A$and θ_B are the concentration of the species A and B in equilibrium then,

$$P_A = C\theta_A \text{ and } P_B = C\theta_B \tag{7}$$

C is proportionality constant relating the pressure of an ideal gas with its concentration.

Now, let's define scaled Langmuir constant as,

$$K_A{}' = CK_A \text{ and } K_B{}' = CK_B \tag{8}$$

The equation (6) can therefore be alternatively written as,

$$< N_A > / \Gamma = F_A = \frac{2K_A{}'\theta_A + 2{\theta_A}^2 {K'_A}^2 e^{-\beta U_{AA}} + 2\theta_A K'_A \theta_B K'_B e^{-\beta U_{AB}}}{1 + 2\theta_A K'_A + {\theta_A}^2 {K'_A}^2 e^{-\beta U_{AA}} + 2\theta_B K'_B + {\theta_B}^2 {K'_B}^2 e^{-\beta U_{BB}} + 2\theta_A K'_A \theta_B K'_B e^{-\beta U_{AB}}} \tag{9}$$

When there are no interactions between the molecules A and B, instead of invoking the cooperative adsorption model, we employ the non-cooperative Langmuir Adsorption model as shown schematically in Figure S4 below.

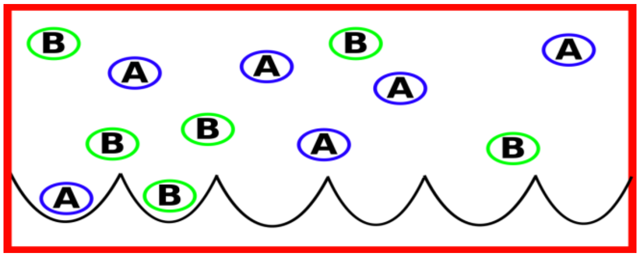

Figure S4. Schematic diagram representing non-cooperative competitive Langmuir adsorption model of two different species A and B. The black semi circles are the adsorption sites which can accommodate only one molecule.

In that case, the expectation value for the number of adsorbed A molecules is

$$< N_A >/\Gamma = F_A = \frac{K_A{}'\theta_A}{1+\theta_A K'_A+\theta_B K'_B} \quad (10)$$

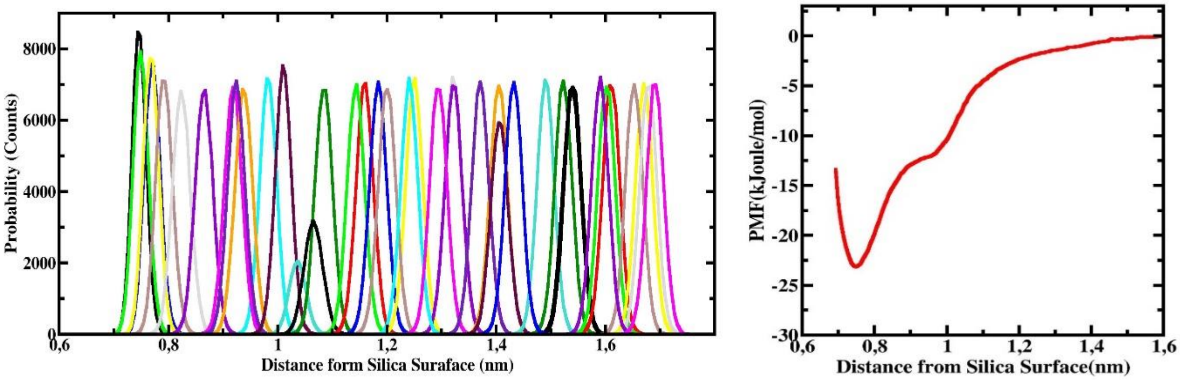

Figure S5. Histograms obtained from the US run with amino acid R and silica (left). The corresponding PMF profile (right).

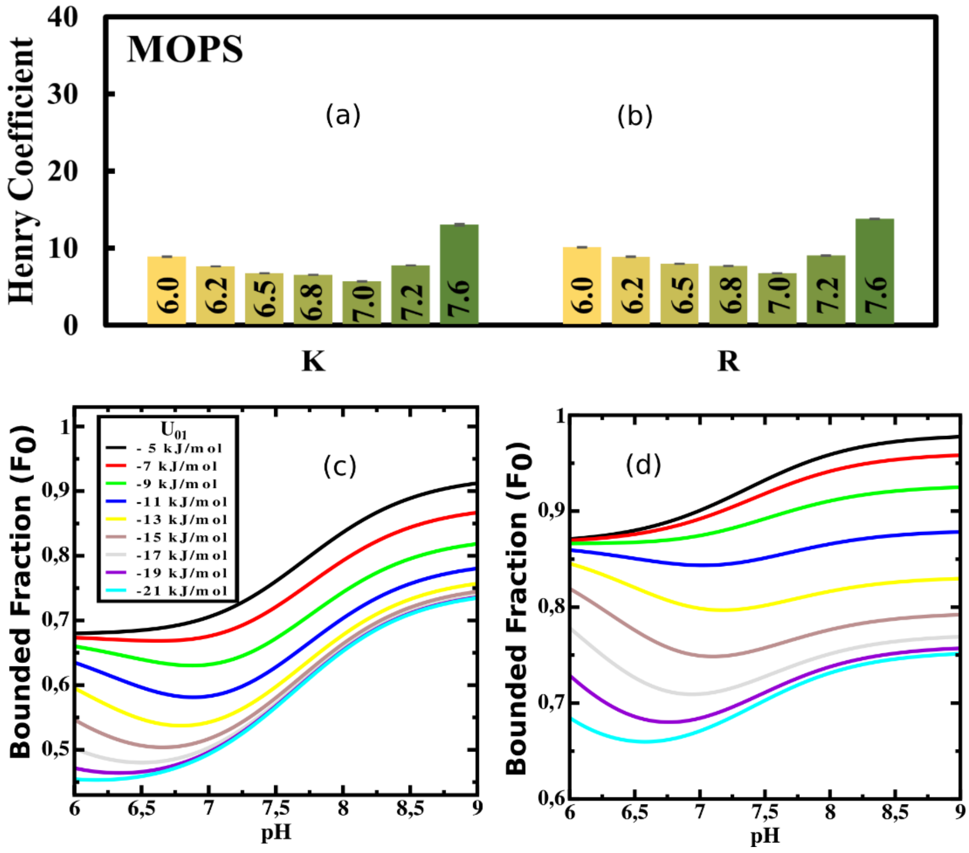

Figure S6: (a/b) Experimentally measured Henry coefficient of K/R as a function of pH in presence of MOPS buffer. (c/d) Fraction of K/R bound to silica for different values of the interaction energy (U_{01}) between K/R and MOPS (Negative) as a function of pH as calculated using multiscale modelling. The experimental Henry coefficient and calculated bounded fraction show qualitatively same behavior for $|U_{01}|$ > 7 kJ/mol (or $|U_{01}|$ > 11 kJ/mol) for K (or R).

Experimental setup

Table S5. Concentration of the amino acids as prepared for the chromatography experiment.

Amino acid	1-letter code	Concentration [mM]
Glycine	G	50
Alanine	A	50
Valine	V	50
Leucine	L	50
Isoleucine	I	50
Proline	P	25
Methionine	M	5
Cysteine	C	10
Asparagine	N	5
Glutamine	Q	10
Aspartic acid	D	15
Glutamic acid	E	25
Serine	S	50
Threonine	T	25
Tyrosine	Y	1
Phenylalanine	F	25
Tryptophan	W	1
Histidine	H	5
Lysine	K	50
Arginine	R	50

The chromatographic column was operated on an Agilent 1100 HPLC system with an UV/Vis detector. Amino acids (AAs) were measured at 210 nm, aromatic AAs additionally at 280 nm. The flow rate was ~12 cm min^{-1} for every run and the injection volume for every AA was 20 µL. The Henry coefficient H was determined with H = k' / φ. Where k' is the retention factor of the AA and φ is the phase ratio of the column. The retention factor is calculated as $k' = (t_R - t_0)/t_0$. Here t_R stands for the retention time of the AA and t_0 for the retention time of a non-interacting tracer in this case 1 g L^{-1} uracil. The phase ratio of the column is calculated with $\varphi = (1-\varepsilon^t)/ \varepsilon^t$. Here ε^t

is the total porosity of the column calculated with the flow rate $\dot{V}$ = 2 mL min^{-1}: $\varepsilon^t = (t_0 * \dot{V}) / V_{column}$. The volume of the column is 0.55 mL.

Table S6. Measured retention times and conversion in Henry coefficient for 18 amino acids in 10 mM TRIS pH 8 on silica gel 60. The total porosity was 0.52 and the phase ratio of the column 0.93. Retention times were measured at room temperature.

Analyte	Retention time [min]				Retention factor k' [-]		Henry coefficient H [-]	
					Ø k'	σ	H	σ
Uracil	0.28	0.28	0.29	0.29				
Glycine	0.29	0.29	0.29	0.29	0.00	0.00	0.01	0.00
Alanine	0.29	0.29	0.29	0.29	0.01	0.01	0.01	0.01
Valine	0.31	0.31	0.31	0.31	0.08	0.01	0.09	0.01
Leucine	0.32	0.32	0.32	0.32	0.11	0.00	0.12	0.00
Isoleucine	0.32	0.32	0.32	0.32	0.12	0.01	0.13	0.01
Methionine	0.31	0.31	0.31	0.31	0.08	0.00	0.09	0.00
Proline	0.37	0.37	0.37	0.37	0.29	0.01	0.31	0.01
Phenylalanine	0.31	0.31	0.31	0.31	0.09	0.00	0.10	0.00
Tryptophan	0.30	0.30	0.30	0.30	0.06	0.01	0.06	0.01
Threonine	0.28	0.28	0.28	0.28	0.01	0.00	0.01	0.00
Serine	0.28	0.28	0.28	0.28	0.03	0.00	0.03	0.00
Cysteine	0.26	0.26	0.26	0.26	0.08	0.01	0.09	0.01
Tyrosine	0.27	0.28	0.28	0.28	0.03	0.00	0.04	0.00
Asparagine	0.28	0.28	0.28	0.28	0.02	0.01	0.02	0.01
Glutamine	0.28	0.29	0.29	0.29	0.00	0.00	0.00	0.00
Aspartic acid	0.20	0.20	0.20	0.20	0.30	0.01	0.33	0.01
Glutamic acid	0.22	0.22	0.23	0.21	0.23	0.02	0.24	0.02
Histidine	0.41	0.42	0.42	0.43	0.48	0.01	0.51	0.02

Table S7. Measured retention times and conversion in Henry coefficient for arginine and lysine in 10 mM TRIS on silica gel 60 for different pH. The total porosity and the phase ration of the column for every buffer run are given in the line of buffer description. Retention times were measured at room temperature.

Analyte	Retention time [min]			Retention factor k' [-]		Henry coefficient H [-]	
				Ø k'	σ	H	σ
**TRIS pH 7.2** (e_t = 0.58, φ = 0.74)							
Uracil	0.32	0.32	0.32				
Arginine	2.22	2.23	2.23	6.05	0.03	8.19	0.04
Lysine	2.03	2.02	2.04	5.41	0.04	7.33	0.05
**TRIS pH 7.6** (e_t = 0.57, φ = 0.77)							
Uracil	0.31	0.31	0.31				
Arginine	3.23	3.24	3.24	9.41	0.02	12.24	0.03

Lysine	2.82	2.83	2.83	8.09	0.03	10.52	0.03
**TRIS pH 8.0** (e_t = 0.56, φ = 0.78)							
Uracil	0.31	0.31	0.31				
Arginine	4.97	4.89	4.92	14.92	0.13	19.18	0.17
Lysine	4.38	4.33	4.33	13.05	0.09	16.77	0.11
**TRIS pH 8.5** (e_t = 0.54, φ = 0.85)							
Uracil	0.30	0.30	0.30				
Arginine	9.64	9.18	9.70	30.90	0.85	36.54	1.01
Lysine	8.84	9.07	8.86	28.94	0.28	34.22	0.33

Table S8. Measured retention times and conversion in Henry coefficient for arginine and lysine in 10 mM MOPS on silica gel 60 for different pH. The total porosity and the phase ration of the column for every buffer run are given in the line of buffer description. Retention times were measured at room temperature.

Analyte	Retention time [min]			Retention factor k' [-]		Henry coefficient H [-]	
				Ø k'	σ	H	σ
**MOPS pH 6.0** (e_t = 0.58, φ = 0.72)							
Uracil	0.32	0.32	0.32				
Arginine	2.67	2.63	2.64	7.25	0.06	10.11	0.09
Lysine	2.35	2.36	2.38	6.38	0.03	8.89	0.04
**MOPS pH 6.2** (e_t = 0.58, φ = 0.71)							
Uracil	0.32	0.32	0.32				
Arginine	2.37	2.35	2.34	6.32	0.04	8.88	0.05
Lysine	2.06	2.09	2.05	5.43	0.04	7.63	0.06
**MOPS pH 6.5** (e_t = 0.59, φ = 0.70)							
Uracil	0.33	0.32	0.32				
Arginine	2.13	2.12	2.12	5.54	0.01	7.96	0.02
Lysine	1.86	1.85	1.84	4.70	0.01	6.75	0.02
**MOPS pH 6.8** (e_t = 0.59, φ = 0.69)							
Uracil	0.33	0.33	0.33				
Arginine	2.04	2.05	2.06	5.31	0.03	7.67	0.04
Lysine	1.78	1.80	1.80	4.51	0.02	6.52	0.03
**MOPS pH 7.0** (e_t = 0.59, φ = 0.70)							
Uracil	0.33	0.32	0.32				
Arginine	1.84	1.85	1.86	4.72	0.04	6.74	0.06
Lysine	1.61	1.61	1.63	4.00	0.04	5.71	0.06
**MOPS pH 7.2** (e_t = 0.59, φ = 0.70)							
Uracil	0.32	0.32	0.32				

Arginine	2.36	2.37	2.38	6.34	0.04	9.02	0.05
Lysine	2.09	2.08	2.09	5.46	0.01	7.76	0.02
**MOPS pH 7.6** (e_t = 0.59, φ = 0.69)							
Uracil	0.32	0.33	0.33				
Arginine	3.41	3.43	3.45	9.57	0.04	13.79	0.05
Lysine	3.28	3.23	3.29	9.05	0.08	13.04	0.12
